# Navigating Scientific Peer Review with ChatGPT: Ally or Adversary?

**DOI:** 10.34172/apb.2024.053

**Published:** 2024-06-29

**Authors:** ArunSundar MohanaSundaram

**Affiliations:** ^1^School of Pharmacy, Sathyabama Institute of Science and Technology, Chennai 600119, Tamilnadu, India.

## Dear Editor,

 ChatGPT (Generative Pre-trained Transformer), a breakthrough innovation by OpenAI) is being touted as a revolutionary tool with immense potential in an array of medical and pharmaceutical research and scientific peer review (SPR) processes.^1,2^ Studies revealed that ChatGPT might act as a complementary tool to the human SPR and aid in expediting the process, reduce reviewer fatigue, and shorten publication timelines.^3,4^ Of note, ChatGPT displayed remarkable competence in providing shrewd feedback, detecting methodological defects, and measuring the article’s impact on the advancement of the respective field, all with a fair inter-rater agreement.^5^

 On the other side, a recent article by Liang et al raised an alarm regarding the perils of using ChatGPT in the peer review process. The study found remarkable alteration using ChatGPT in nearly 17% of the peer-review reports.^6^ The researchers have analyzed about 146 000 peer reviews submitted to the AI conferences (pre- and post-launch of ChatGPT) and found a remarkable upsurge in the use of certain buzzword adjectives like versatile, meticulous, intricate, etc. (the telltale signs of ChatGPT-written text) in the review reports.

 ChatGPT has limited utility in the SPR due to a lack of transparency in training data and decision-making process, issues with the reproducibility of review reports, inability to justify the recommendations, potential biases, and AI hallucinations (generation of fake/non-existing references for writing and reviews). Besides, lack of contextual expertise, missing human connect (iterative fine-tuning, personal interaction and collaboration between reviewers and authors, and nuanced, context-based considerations), lack of accountability and incapability in image interpretation (in free-version; ChatGPT 3.5) are additional challenges.^5^ Hence, performing fully automated ChatGPT-based SPR is far from practical implementation.^3,4^ Besides, there is a possibility of unprecedented repercussions in defining and shaping the scholarly communities when using ChatGPT for the SPR. However, regular training with appropriate, unbiased datasets, periodical audits and mitigation of model biases might improve the capabilities of ChatGPT.

 Taken together, ChatGPT is a quintessential tool for advancing the SPR process. While it is good to leverage this technology in the AI-driven SPR process, continuous improvement, cautious implementation, and constrained, human-supervised processing is obligatory for disseminating high-quality and ethical scientific research.

## Competing Interests

 None.

## Ethical Approval

 Not applicable.
